# Motivations and fears driving participation in collaborative research infrastructure for animal tracking

**DOI:** 10.1371/journal.pone.0241964

**Published:** 2020-11-20

**Authors:** Tara L. Crewe, Dave Kendal, Hamish A. Campbell

**Affiliations:** 1 Research Institute for the Environment and Livelihoods, Charles Darwin University, Darwin, Northern Territory, Australia; 2 Geography and Spatial Sciences, University of Tasmania, Sandy Bay, Tasmania, Australia; University of Bucharest, ROMANIA

## Abstract

Anthropogenic derived environmental change is challenging earth’s biodiversity. To implement effective management, it is imperative to understand how organisms are responding over broad spatiotemporal scales. Collection of these data is generally beyond the budget of individual researchers and the integration and sharing of ecological data and associated infrastructure is becoming more common. However, user groups differ in their expectations, standards of performance, and desired outputs from research investment, and accommodating the motivations and fears of potential users from the outset may lead to higher levels of participation. Here we report upon a study of the Australian ornithology community, which was instigated to better understand perceptions around participation in nationally coordinated research infrastructure for detecting and tracking the movement of birds. The community was surveyed through a questionnaire and individuals were asked to score their motivations and fears around participation. Principal Components Analysis was used to reduce the dimensionality of the data and identify groups of questions where respondents behaved similarly. Linear regressions and model selection were then applied to the principal components to determine how career stage, employment role, and years of biotelemetry experience affected the respondent’s motivations and fears for participation. The analysis showed that across all sectors (academic, government, NGO) there was strong motivation to participate and belief that national shared biotelemetry infrastructure would facilitate bird management and conservation. However, results did show that a cross-sector cohort of the Australian ornithology community were keen and ready to progress collaborative infrastructure for tracking birds, and measures including data-sharing agreements could increase participation. It also informed that securing initial funding would be a significant challenge, and a better option to proceed may be for independent groups to coordinate through existing database infrastructure to form the foundation from which a national network could grow.

## Introduction

The sharing and integration of research data between groups, institutes, and organisations has the potential to produce unique and important findings for science and humanity [1–6]. It has been demonstrated that researchers that share research data benefit from increased citation rates [7], increased opportunities for collaboration and publication [8], and the standardization, archival, increased visibility, and re-use of their data [9–12]. Whilst many fields of science have benefited from structured procedures for the sharing and re-use of data, there still persists serious concerns in the ecological research community as to its practice [13, 14]. These include fears of data misuse, publication of the locations of at-risk species, interference with long-term monitoring experiments [14], use of data without consent or citation, and being gazumped to significant findings [8, 13]. Nevertheless, ecological-based data portals to enhance data-sharing, discoverability and reuse are growing in acceptance and uptake by the research and natural resource management communities [12, 15–18].

A step beyond the sharing of data is the funding and development of collaborative research infrastructure [15, 19–21]. Research that requires expensive infrastructure, such as astronomy and physics, have a long history of collaborative research infrastructure, which necessitates the production of research papers with many associated authors [22]. However, the concept is relatively new to ecologists. One field that is leading the way in ecology is animal biotelemetry [3, 23]. With biotelemetry, electronic devices are attached to free-ranging animals, and the sharing of infrastructure typically takes the form of field-based receivers that detect and log the presence of the electronically tagged animals when within range of a static receiver [15, 21, 24]. Such ‘node-based’ networks rely on the inter-compatibility of data collected at each node to monitor animal movements at fine to broad temporal and spatial scales. At the heart of these node-based networks is a centralized data portal, which allows all transmitters deployed to be detected by all receivers throughout the network, and some type of governance structure to ensure that transmitter ID’s are not duplicated. In aquatic environments, (Australia: Integrated Marine Observing System [25]; North America: Ocean Tracking Network [20], Atlantic Cooperative Telemetry [26], California Fish Tracking Consortium (http://cftc.metro.ucdavis.edu/), Florida Acoustic Cooperative Telemetry (https://secoora.org/fact/); and South Africa: Acoustic Tracking Array Platform [27]), node-based networks have been integral in informing the movements, space use, meta-population structure, interactions, habitat preferences, and migration routes of aquatic animals [3, 25]—with this information being applied to the conservation and management of aquatic species [23, 28].

The development of multi-user shared node-based networks of biotelemetry receivers in terrestrial environments has not experienced the same pace of growth as in aquatic environments. The reasons for this are unclear but worth discovering because the global expansion of terrestrial node-based networks has the potential to provide unique insights into the movements, habitat hot-spots, and migration pathways for birds, bats, and insects. This is particularly true for flying animals that are too small to carry the current suite of satellite-based telemetry devices, for research questions that require monitoring over extended periods of time, and/or when the retrieval of the telemetry device is not possible. The ability to assemble automated terrestrial VHF-receivers from off-the-shelf components is now incredibly cheap and has led to a growth in online community support for VHF receiver construction and development (e.g., sensorgnome.org). This presents the opportunity for nearly anyone to build and register a coded-VHF detection node within a collaborative terrestrial network, providing citizen science engagement opportunities. This community contribution of receivers can greatly increase the spatial coverage of a network and reduce the costs to researchers tagging the animals [15, 29–31].

The Motus Wildlife Tracking System (hereafter ‘Motus’; https://motus.org) [15], developed in North America between 2012–2015, is the first example of a broad-scale, international collaborative research network that uses VHF receivers to track the movements of terrestrial organisms. The network has its roots in a regional Eastern North American network developed in 2012 by Acadia University with numerous partners. Following federal funding grants to develop a centralized database portal and expand the receiver array in eastern Canada, the growth of Motus throughout the Americas and internationally has been a primarily grassroots movement, whereby as more organizations embarked on projects that added receivers to the network, the added benefits of contributing became more apparent. This has been followed by scientific research output and findings that would not have otherwise been possible [2, 32]; over 100 publications are currently listed on the Motus publications page (https://motus.org/data/publications). Currently there exists no comparable collaborative node-based infrastructure for the collection of terrestrial animal movement data in Australia [24], despite centralised governmental funding for a range of national coordinated research infrastructure to monitor the environment and biodiversity (Terrestrial Ecosystem Resource Network [33], Integrated Marine Observing System [21, 25], Atlas of Living Australia [34]). An understanding of how potential user communities perceive data and infrastructure sharing, and what factors drive or inhibit participation, would help guide planning and development of an Australian network to maximize uptake and ensure the network’s long-term viability and success.

This paper documents a study of the Australian ornithological community to assess their motivations and fears of using shared research infrastructure to track the movements and migration of birds, and their willingness to participate in its development. Specifically, we aimed to understand if motivations and fears varied with age (younger people are more favourable of data sharing [11]), with their association with avian research, and/or with their experience in the field of animal biotelemetry. We discuss results in the context of how user support may be maximized by addressing the perceptions of specific user groups during the infrastructure planning and development stage. The approach and results are of relevance to, and may advise, those wishing to develop coordinated shared research infrastructure.

## Methods

### Survey and target audience

An anonymous survey was developed to identify members of the Australian ornithological community’s interest in, and perceptions of, a nationally coordinated biotelemetry network for tracking bird movements. The survey was approved by the Charles Darwin University human ethics committee (project H19045), and participants opted in and provided consent by submitting a survey; data were collected and analysed anonymously. The survey (S1 Survey) included questions designed to inform the characteristics of the participant and their motivations and fears towards a) a nationally coordinated network to track the broad scale movements of birds, and b) sharing data with a nationally coordinated database. In each case, we asked a series of questions that allowed individuals to score from 0 (no benefit or risk) to 10 (great benefit or risk) their perception of 1) individual motivations (e.g., publication and collaboration opportunities, financial gains) and 2) fears (e.g., data sharing, technical and analytical support, financial costs). In total, 28 items were used to measure perceived motivations and fears (Questions 15–18; S1 Survey). Similarly, to better understand how individuals might interact with a nationally coordinated network, we asked individuals to score from 0 (unlikely) to 10 (very likely) their likelihood of interacting with a nationally coordinated network through data collection or network development (Question 21; S1 Survey).

Both a paper and electronic version of the survey were distributed. The paper version was distributed during a workshop at the Australasian ornithological bi-annual conference in 2019 on ‘Building a collaborative research network to track bird movement through Australasia’. The electronic version was distributed using a snowball sampling method whereby an email was distributed to all registered attendees of the Australasian Ornithological Conference (approx. 350 individuals) and to research-related groups associated with BirdLife Australia. Individuals were asked to distribute the survey to colleagues; the number of individuals whom received the survey is therefore not known but presumed to be a significant proportion of the Australian ornithological community.

Data from paper and electronic surveys were combined for all analyses. It is possible that some individuals submitted both paper and electronic surveys. Because both surveys were anonymous, paper and electronic surveys were compared and 5 paper surveys were removed from consideration because they had an electronic duplicate.

### Data analysis

#### Classifying survey participants

To provide sufficient power to test the influence of age, employment and biotelemetry experience on users motivations and fears it was necessary to group the age of participants into cohorts. Selected age cohorts were consistent with the groupings from a previous study on the perceptions of data sharing in the sciences [8]. The various employment roles provided from the survey were classified into those of ‘research’-focused or ‘non-research’ focused positions, where research-focused roles included undergraduate and higher degree by research students, Post-Doctoral fellows, research assistants, and individuals in faculty, government, NGO, or industry scientist positions. Amateur ornithologists, Citizen Scientists, conservation or resource managers, environmental consultants, and any other employment roles were considered non-research roles. Finally, to provide a measure of a participant’s degree of understanding around the logistics and cost of collecting biotelemetry data, users were asked to provide their biotelemetry experience in number of years; these were grouped into cohorts of ‘0’ years (no biotelemetry experience), ‘1–9’ years, and ‘10+’ years.

#### Assessing motivations and fears of using shared research infrastructure

We first reduced the dimensionality of the data from 28 by applying a Principal Components Analysis (PCA) using the *principal* function of the *psych* package [35] in R [36]. PCAs are frequently used in psychological research to identify groups of questions where individuals responded similarly [37], resulting in fewer ‘components’ that can be used to describe the variability in the data. A scree plot was used to determine the number of components to include in the final model, and reliability of those components was assessed using Cronbach’s alpha through the *alpha* function in the *psych* R package [35]. In other words, questions where individuals responded similarly were grouped into components; to test whether individual responses for each component varied with age, research role, and biotelemetry experience, we first took the average of the ranked responses for each individual across questions loading on each component, and then for each component, performed AIC model selection to test which of a series of hypothesized linear regression models had strongest support. Linear regression models were run in R [36] where the average score for each individual was the response variable, and combinations of age group, research role, and biotelemetry were explanatory variables. Model fit was assessed using residual plots. We also tested for similarity in mean responses among components using a Spearman rank correlation matrix using the *chart*.*Correlation* function of the *PerformanceAnalytics* R package [38].

#### Assessing likelihood of participation in shared research infrastructure

The willingness of respondents to participate in the various aspects of the development of nationally coordinated infrastructure was assessed using the same protocol as above, where a PCA was applied to the 10 responses in Question 21 (S1 Survey). Data wrangling in R was performed using the *tidyverse* [39] and *dplyr* [40] packages; plots were created using *ggplot2* [41].

## Results

In total, 105 independent surveys (15 paper, 90 electronic) were submitted. Of those, 77 and 80 individuals answered all questions for the PCAs on network motivations and fears and network participation, respectively. Responses were received from individuals from across all Australian states and territories, with 8 surveys submitted by individuals from New Zealand. Surveys from New Zealand were retained in all analyses as respondents were from the greater Australasian region.

### Classifying survey participants

**Fig 1:**
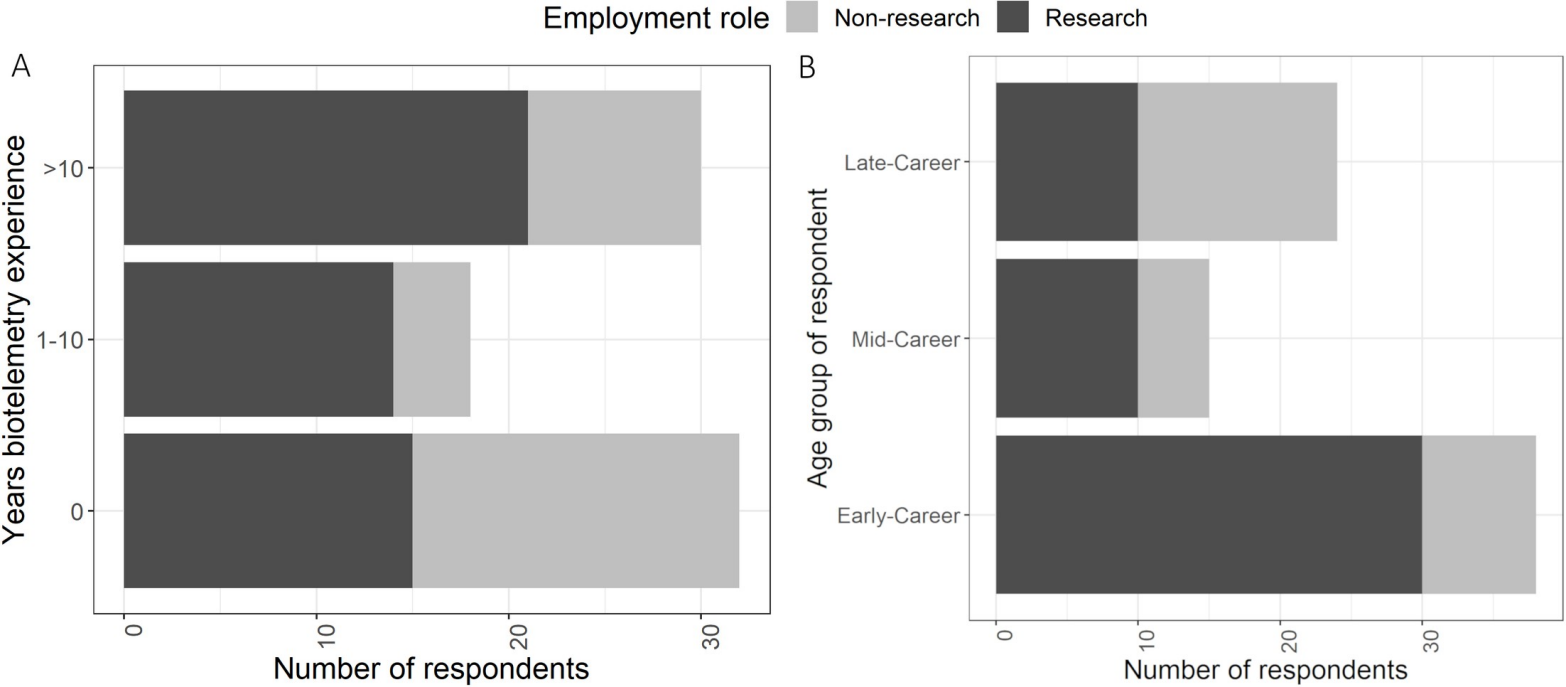
Distribution of respondents among A) biotelemetry experience, B) age group, with respondents classified as either in research-focused (dark grey) or non-research-focused (light grey) employment.

Of the participants that stated a research interest, 74% had interest in the study of the movement and/or migration of birds, and approximately half of participants did not have previous experience in the use of animal biotelemetry equipment; individuals in research-focused positions (n = 50) tended to have more biotelemetry experience than individuals in non-research focused positions (n = 30; Fig 1A). Of the 80 individuals included in the analysis, 39 individuals were classified as early career (ages 18–39), 15 as mid-career (ages 40–49), and 26 as late career (ages 50+; Fig 1B). Overall, participants scored highly that a national network would improve the conservation and management of Australia’s avifauna (mean score = 8.52/10, se = 0.88, 95% CL = 6.80, 10, where 10 meant ‘strongly agree’; Question 13, S1 Survey) and contribute to increased knowledge of bird spatial movement and migration (mean score = 9.15/10, se = 0.95, 95% CL = 7.29, 10, where 10 meant ‘strongly agree’; Question 14, S1 Survey). Sixty-three percent of respondents were more likely to share data if a strict data sharing policy were in place (Question 20, S1 Survey).

### Model selection: Motivations and fears of using shared research infrastructure

**Fig 2:**
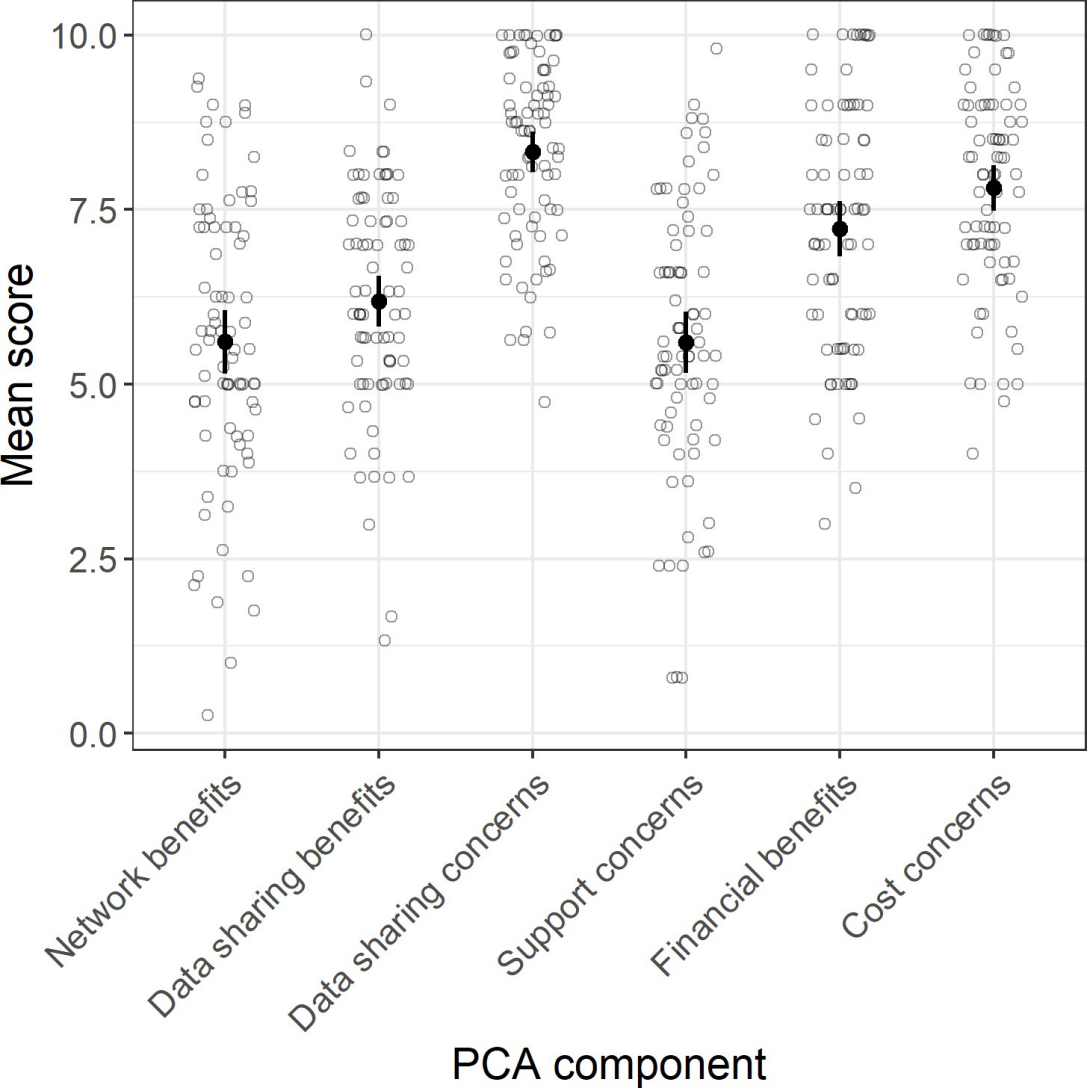
Individual scores (grey) and mean ± 95% confidence limits of scores across respondents (black) for each of six PCA components. A score of 0 suggests respondents perceived either no benefit or no concern, and a score of 10 suggests respondents perceived either great benefit or great concern.

PCA factor loadings showed the 28 items could be grouped into six components (Table 1): 1) ‘Network Benefits’ included questions pertaining to individual benefits such as increased collaboration, research, funding and publication opportunities, and altruistic benefits including increasing knowledge of animal movements at broader spatial and temporal scales, and improved species management and conservation; 2) ‘Data Sharing Concerns’ included concerns around improper re-use of data, data reuse without permission, lack of incentives to share, and sensitivities around the publication of species locations; 3) ‘Data Sharing Benefits’ included data standardization, safe archival, and increased data discoverability, access and reuse; 4) ‘Support Concerns’ included concerns around gaining government and financial support for a network, as well as technical and analytical support to participate in a network; 5) ‘Cost Concerns’ included concerns around the financial cost of maintaining and participating in a network (e.g., user fees), and the risk that ongoing technological advances in position-logging geographic positioning system (GPS) tracking devices will negate the need for a node-based network; and 6) ‘Financial Benefits’ included the reduced operational costs to researchers when participating in a shared network (Table 1, Fig 2). Cronbach’s alpha scores ranging between 0.68 and 0.90 for each component suggested good model fit (Table 1). Mean score for all components was greater than 5 (Fig 2), suggesting participants recognized both the benefits and challenges of developing a national network and of sharing data within that network. In general, individuals showed greatest concern with sharing data and with the costs associated with developing and maintaining a network (including investment into a costly autonomous network in light of ongoing technological advances in GPS; Fig 2).

**Table 1:** Survey participants ranked perceptions of various 1) motivations and 2) fears of a) participating in a nationally coordinated network and b) sharing data with a nationally coordinated database.

Perception	a. Nationally coordinated network	b. Nationally coordinated database	PC	PC Label	Factor loadings
1. Motivations	• Publication opportunities		2	Participation motivations (0.90)	0.72
	• Collaboration opportunities	• Collaboration opportunities			0.75, 0.75
	• Funding opportunities				0.54
	• Improved conservation and management	• New research perspectives			0.80, 0.82
	• Increased knowledge of bird movements over broad spatial and temporal scales	• Broader spatial/temporal scales of research			0.86, 0.82
		• Standardized data/metadata	5	Data sharing motivations (0.68)	0.73
		• Safe data storage/increased data permanence			0.76
		• Data discoverability and reuse			0.74
	• Reduced operational costs	• Reduced operational costs	6	Financial motivations (0.74)	0.76, 0.62
2. Fears	• Technological support		3	Support fears (0.85	0.80
	• Analytical support				0.81
	• Gaining government/granting agency support				0.61
	• Non-Australian organization as network host	• Non-Australian organization as database host			0.72, 0.63
	• User fees		4	Cost fears (0.84)	0.73
	• Cost of infrastructure maintenance				0.70
	• Ongoing technological advances will supersede the need for a telemetry network				0.57
	• Concerns with sharing data	• Lack of incentive/reward for sharing (e.g., co-authorship, proper citation)	1	Data sharing fears (0.90)	0.60, 0.83
		Time to publish before reuse			0.79
		• Sensitivities around species locations			0.76
	• Data reuse guidelines	• Data reused without permission			0.90
		• Inappropriate reuse or interpretation of data			0.86

For each motivation or fear, individuals scored from 0 (no perceived motivation/no perceived fear) to 10 (great perceived motivation/high perceived fear). Principle components analysis was used to reduce the dimensionality of the perceived motivations and fears by grouping questions where individuals responded similarly; ‘PC’ gives the principle component on which each question weighted most heavily (see ‘Factor loadings’), showing which motivations and which fears were grouped together by the PCA. ‘PC Label’ classifies the PCs into general themes and Cronbach’s alpha for each component is shown in parentheses.

Significant correlation between network benefits and data sharing benefits (0.50, p < 0.001; S1 Fig) suggests that individuals who recognized the benefits of a nationally coordinated network also recognized the benefits of sharing data within a network; these same individuals also tended to have greater concerns with regards to the logistical (technical, analytical and financial) support required to participate in a network (0.61, p < 0.001; S1 Fig). Strong correlation between financial benefits and cost concerns (0.70, p < 0.001; S1 Fig) suggests that while sharing operational costs across a network was viewed as beneficial, there remained concerns with regards to the financial cost of developing and supporting a national network, and the potential cost associated with continued miniaturization of GPS technology. Individuals concerned about network costs were also more concerned about data sharing (0.61, p < 0.001; S1 Fig).

Results of AICc model selection for each PCA component are shown in S1 Table, and model averaged estimates for all competing models (i.e., models with ΔAICc ≤2) for each PCA component in Table 2. Confidence intervals for each model-average effect included zero for the Network Benefits, Data Sharing Benefits, Support Concerns, and Cost Concerns components, which suggests that effects of age, research role and biotelemetry experience on each component score were not strongly supported by the data, i.e., respondents tended to score similarly on those components regardless of background.

**Table 2:** Model averaged estimates for competing models (ΔAICc < = 2; S1 Table) for each PCA component, where component score was the dependent variable and independent variables included age group (reference level = early career), employment role (reference level = non-research) and/or years of biotelemetry experience (reference level = no experience).

Component	Parameter	Estimate	SE	LCL	UCL
Network benefits	Age group: mid-career	-0.44	0.62	-1.67	0.78
	Age group: late-career	-0.80	0.54	-1.86	0.26
	Employment role: Research	-0.16	0.51	-1.16	0.84
	Biotelemetry experience: 1-9yrs	0.00	0.53	-1.03	1.03
	Biotelemetry experience: >10yrs	-0.35	0.62	-1.57	0.88
Data sharing benefits	Biotelemetry experience: 1-9yrs	-0.22	0.43	-1.05	0.62
	Biotelemetry experience: >10yrs	0.29	0.51	-0.70	1.29
	Age group: mid-career	-0.36	0.51	-1.36	0.64
	Age group: late-career	0.03	0.43	-0.81	0.88
	Employment role: Research	-0.04	0.39	-0.81	0.73
Data sharing concerns	Employment role: Research	0.21	0.32	-0.42	0.83
	Biotelemetry experience: 1-9yrs	0.11	0.33	-0.54	0.76
	**Biotelemetry experience: >10yrs**	**-0.88**	**0.39**	**-1.64**	**-0.11**
Support concerns	Employment role: Research	-0.54	0.46	-1.45	0.37
Financial benefits	Employment role: Research	0.29	0.43	-0.56	1.13
	Biotelemetry experience: 1-9yrs	0.33	0.45	-0.55	1.20
	**Biotelemetry experience: >10yrs**	**-1.05**	**0.53**	**-2.09**	**-0.02**
Cost challenges	Biotelemetry experience: 1-9yrs	0.27	0.38	-0.47	1.02
	Biotelemetry experience: >10yrs	-0.11	0.45	-1.00	0.77
	Employment role: Research	0.21	0.35	-0.48	0.89

Effects with confidence limits that exclude zero (bold) were considered strongly supported by the data.

**Fig 3:**
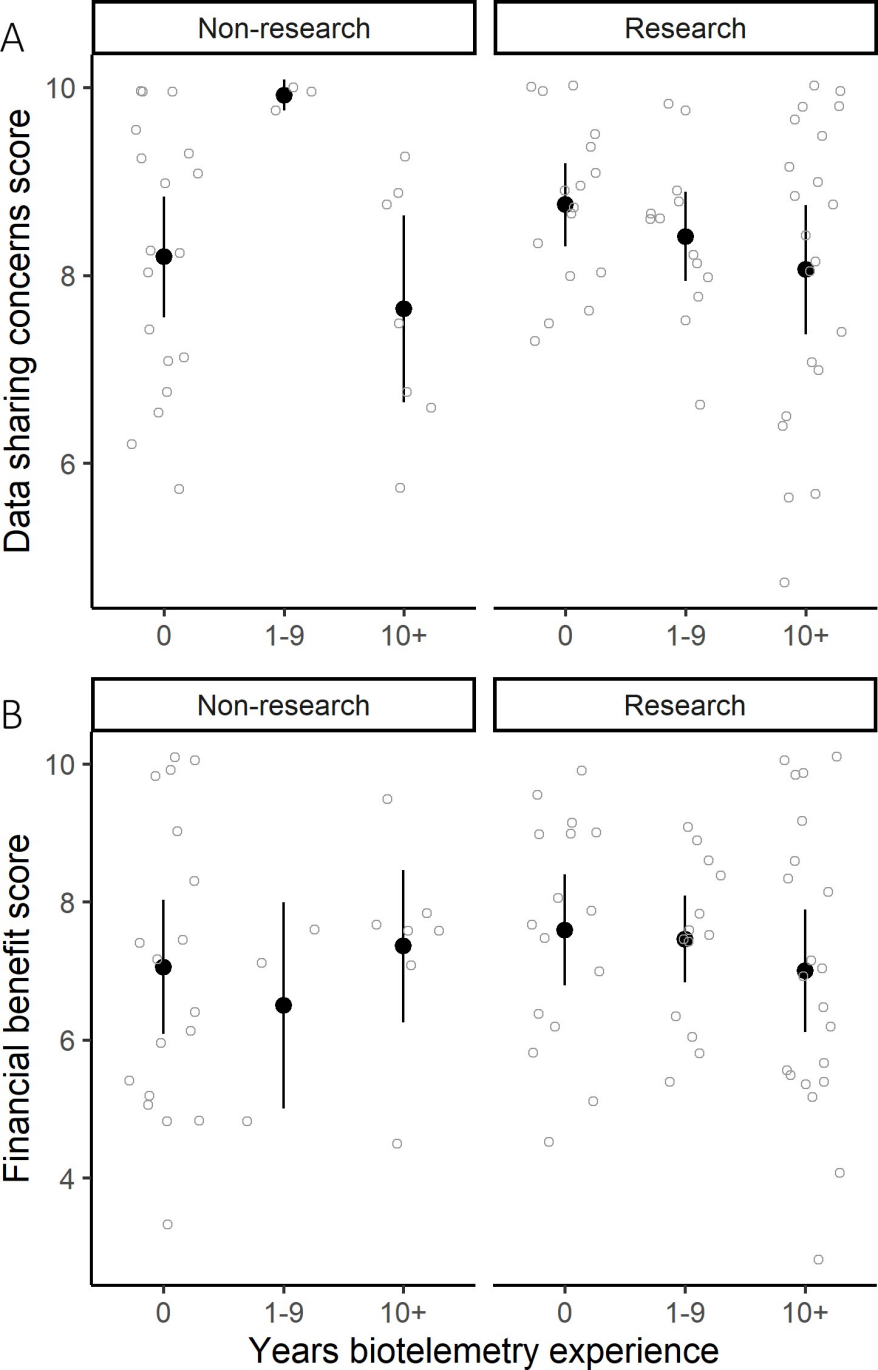
Variation in individual scores (light grey) and mean ± 95% confidence limits of respondent scores (black) by employment role and years of biotelemetry experience for A) the data sharing concerns component, and B) the financial benefits component.

For the Data Sharing Concerns component, two competing models supported effects of biotelemetry experience and employment role (S1 Table). Individuals in research roles tended to be more concerned about sharing data with a network, and individuals with 10 or more years’ biotelemetry experience were less concerned about sharing data, although the distribution of scores for researchers with 10+ years biotelemetry experience suggests that some did have strong data sharing concerns. The overall lower data sharing concern for individuals with 10+ years biotelemetry experience was strongly supported by the data, with confidence limits for the model-averaged estimate that excluded zero (Table 2, Fig 3A).

For the Financial Benefits Component, two competing models supported that individuals in research-based positions perceived greater financial benefits to participation in a network and individuals with 10 or more years of biotelemetry experience perceived fewer financial benefits of a network (S1 Table, Fig 3B). The latter effect was strongly supported by the data, with 95% confidence limits that excluded zero (Table 2).

### Model selection: Willingness to participate in shared infrastructure development

PCA factor loadings grouped survey questions relating to potential network interaction into two components: 1) potential contribution to network development through the development of analytical methods or receiver software and hardware, and 2) potential contribution to data collection through the deployment of receivers and/or tracking devices and providing open data access (Table 3). Cronbach’s alpha scores of 0.85 and 0.83, respectively, suggested good model fit.

**Table 3:** Survey participants scored perceptions of how they might interact with various aspects of a nationally coordinated network, where responses ranged from 0 (not likely to participate) to 10 (high likelihood of participation).

Perception: Network Participation	PC	PC Label	Factor Loading
Perform meta-analyses of data	1	Network Development (0.85)	0.79
Develop new analytical methods			0.82
Relate movement data to landscape Covariates			0.72
Contribute to receiver development			0.74
Relate movement data to physiological data			0.58
Deploy transmitters: one species	2	Data Collection (0.83)	0.81
Deploy transmitters: multiple species			0.78
Deploy receivers: 1 location			0.80
Deploy receivers: multiple locations			0.72
Provide open data access			0.54

Principle components analysis was used to reduce the dimensionality of the perceived network interactions by grouping questions where individuals responded similarly; ‘PC’ gives the principle component on which each question weighted most heavily (see ‘Factor loadings’), showing which aspects or participation were grouped together by the PCA. ‘PC Label’ classifies the PCs into general themes, with Cronbach’s alpha, a measure of fit for each PC, shown in parentheses.

**Fig 4:**
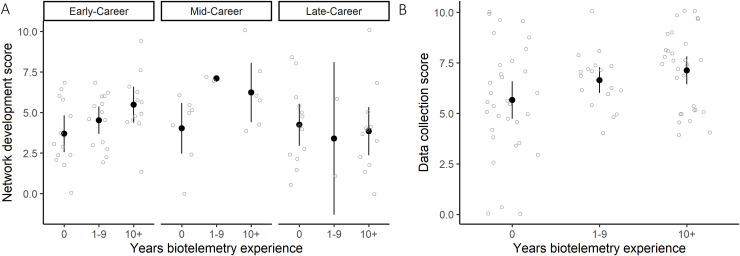
Variation in individual scores (light grey) and mean ± 95% confidence limits of respondent scores (black) A) by age group and years of biotelemetry experience for the PCA component relating to potential involvement in the development of a network by contributing to analytical or technological development or performing meta-analyses on the data, and B) by years of biotelemetry experience for the PCA component relating to potential involvement in collecting data using the network, by deploying transmitters, receivers, or open data.

For the Network Development component, all explanatory variables (age group, biotelemetry experience and employment role) were included in the top five competing models (ΔAIC ≤ 2) (S2 Table). In all cases confidence limits for the model-averaged effects included 0, which suggests the data did not strongly support differences among groups (Table 4). However, confidence limits only marginally included zero for the effect of >10 years biotelemetry experience; results suggested an individual’s concern around data sharing and probability of contributing to infrastructure development increased with biotelemetry experience (Table 4, Fig 4A).

**Table 4:** Model averaged estimates for competing models (ΔAICc < = 2; S2 Table) for each PCA component related to potential individual interaction with a national biotelemetry network.

Component	Parameter	Est	SE	LCL	UCL
Network development	Age group: mid-career	0.78	0.68	-0.54	2.11
	Age group: late-career	-0.55	0.58	-1.68	0.58
	Biotelemetry experience: 1-10yrs	0.69	0.66	-0.60	1.98
	*Biotelemetry experience*: *> 10 years*	*1*.*00*	*0*.*56*	*-0*.*09*	*2*.*09*
	Employment role: research	-0.16	0.55	-1.25	0.93
Data collection	Biotelemetry experience: 1-10yrs	0.98	0.64	-0.27	2.24
	**Biotelemetry experience: > 10 years**	**1.47**	**0.55**	**0.39**	**2.56**

In all models, component score was the dependent variable and independent variables included age group (reference level = early career), employment role (reference level = non-research) and/or years of biotelemetry experience (reference level = no experience). Effects with confidence limits that exclude zero (bold) were considered strongly supported by the data; effects with confidence limits that were largely positive or negative (italic) were considered marginally supported by the data.

For the data collection component, only one model was considered a competing model and included biotelemetry experience as an independent variable. Probability of contributing to data collection increased with years of biotelemetry experience, and this effect was strongly supported by the data, with 95% confidence limits that excluded zero (Table 4, Fig 4B).

## Discussion

Our study found that the Australasian ornithological community is a highly active research group comprised of academia, industry, government and NGO’s. They are distributed across a wide range of age groups and located throughout the continent. The community is highly engaged in the use of animal biotelemetry equipment to track bird movement and migration, but until now the sharing of research infrastructure and data has been very limited. There was however strong support for a national collaborative network of VHF-radio towers to track the spatial movement and migration of birds, similar to what exists in North America and Europe. A high proportion of survey participants believed such a network would improve the conservation and management of Australian birds. Nevertheless, participants expressed strong concerns around securing the necessary finances to build a receiver network at the continental scale. Interestingly, individuals that scored high for this concern also showed strong support for the network, with scores for both increasing with experience in deploying biotelemetry devices on birds. This may reflect a ‘you don’t know what you don’t know’ ideology, whereby less experienced participants do not fully appreciate the challenges in gaining financial support for conservation-outcome driven research. Still, the survey results showed that if the initial outlay costs for the construction of the network could be overcome, then there would be a high degree of participation by both experienced and unseasoned researchers. These would be spread across all the age groups throughout Australasia, and by scientific researchers, natural resource managers and conservation practitioners. Here we explore the various strategies to achieve this.

**Fig 5:**
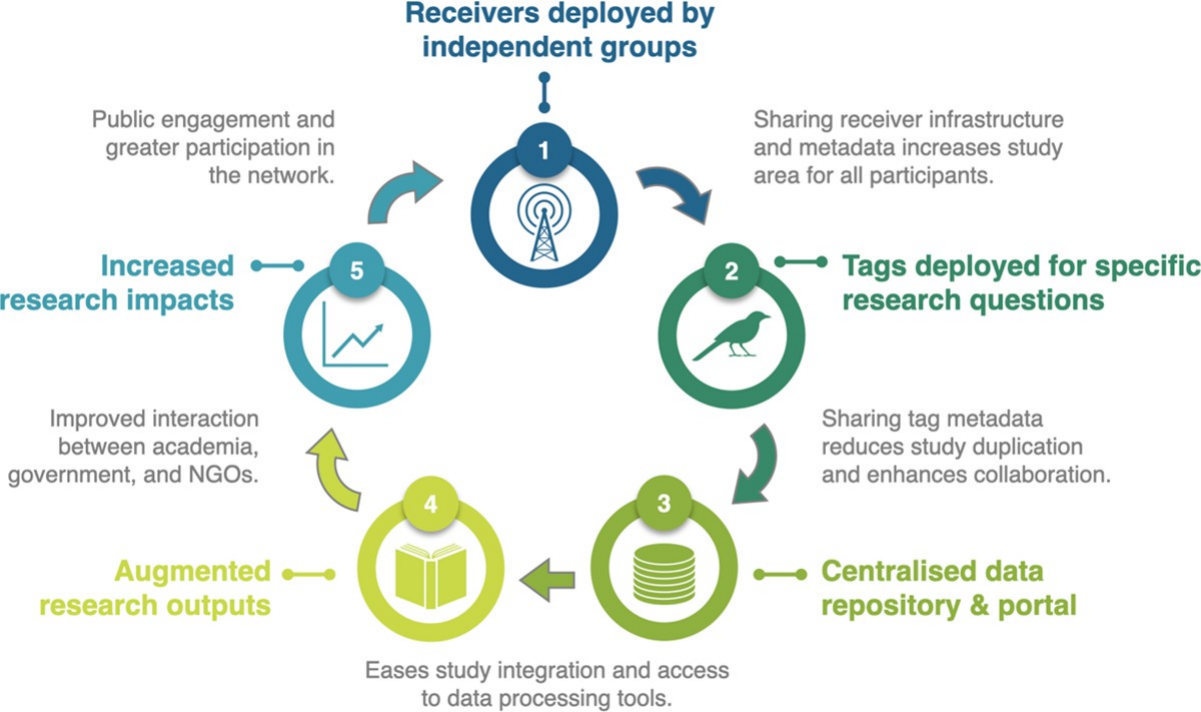
Schematic representation of the components and benefits of node-based collaborative research infrastructure for detecting and tracking the movements of animals.

In the Americas, the construction of a significant portion of the initial Motus VHF receiver infrastructure (100+ stations) was initially funded in 2013 by a large government infrastructure grant awarded to a group of Universities and a non-government, non-profit organization, Birds Canada. Further government, private foundation/donor, and investment by Birds Canada supported the development of the centralized data portal and coordination framework [15]. Since 2013, the network has grown through primarily grassroots to include more than 200 independent research projects comprising 600+ receiver stations across North America. This demonstrated a high motivation by the ornithology community to participate in collaborative research infrastructure once the ground work was done and the benefits were tangible. As more users participated, the geographic coverage of the network increased, and now spans the Americas and is increasingly global (https://motus.org/data/receiversMap). In turn, collaborators benefit from lower costs, increased spatial scope, shared analytical and education resources, better coordination and research outcomes. Thus, while participants of our survey were concerned about garnering funding support for the development of an Australasian VHF receiver network, a grassroots network growth that requires less large-scale and up-front investment may be a plausible model (Fig 5). Particularly if initial costs of database development can be avoided by using already existing database, coordination, and technological infrastructure through Motus. Developing the capacity to share those data with an already existing Australian portal, e.g., the Atlas of Living Australia [34], would ensure data are archived and accessible through a national portal. Housing data within a host country can increase the probability that individuals will contribute data to an open-access database [10, 19].

However, under grassroots growth, the location and density of receivers relies heavily on the geographic distribution of researchers and their study organisms, and the length of time the research and associated receivers are active. This can limit how the data can be used at broader spatial scales [15]. On a continent as large and relatively unpopulated as Australia, relying on grassroots growth may result in temporally and spatially inconsistent geographic coverage of receivers [25]. This issue could be resolved by the deployment of receivers at strategically chosen locations, specifically to facilitate connectivity between independent projects and enable detection of broad-scale animal movements [24]. In North America, regional coordination networks have also emerged, establishing and maintaining significant ‘permanent’ community infrastructure. A similar approach to this has already been undertaken in Australia to track the movements of aquatic animals (IMOS—Animal Tracking Facility (ATF); [25]). In the ten years the IMOS-ATF has been operational it has collected 49.6 million valid detections from 3,777 transmitters deployed on 117 marine species, with distances travelled ranging from a few to thousands of kilometres [25]. Connectivity between regions was only made possible by the joint contribution of the IMOS-ATF infrastructure (40%) and researcher-funded receivers (60%).

The typical concerns expressed by ecologists around the sharing of research data were also expressed by participants of this survey [10, 11, 14]. However, individuals experienced in the collection of biotelemetry data were less concerned about data sharing and more likely to contribute to the development of a network. Previous surveys of animal biotelemetry researchers found that older respondents were less likely to share data [11]. We did not find an effect of age on data sharing concerns in this survey, suggesting that experience with and knowledge of a system played a greater role than age in an individual’s willingness to share data in this case. As two-thirds of respondents were more likely to share data if a strict data sharing policy were in place, offering and advertising clear data sharing and participation policies could reduce fears around data sharing and promote participation among individuals that are showing reluctance to participate. Incentives such as data embargoes and data sharing policies will also ensure individuals are aware of options to maintain control over the sharing and reuse of their data. To test whether experience with a collaborative network does reduce data sharing concerns, individuals already participating in and experienced with a collaborative network (e.g., Motus in North America [15]) could be surveyed and results compared with those described here.

Survey respondents also had concerns about the ongoing development of alternative tracking (GPS) technologies. Whilst ongoing and rapid technological advancement is a concern for any technology-based expensive research infrastructure, VHF radio-telemetry transmitters remain the only devices small enough to track the position of a large proportion of small mobile animals and insects over broad spatial and temporal scales. Tracking the movements of small bodied animals is important because many are of conservation concern, others provide ecosystem services, whilst others are agricultural pests or pose harm to human health. The International Cooperation for Animal Research Using Space initiative (www.icarusinitiative.org) uses an antenna attached to the international space station to detect transmitters from small bodied tagged animals and may supersede the requirement for ground-based stations. However, this only has a single point of data retrieval, which requires cutting edge space technology, international collaboration and huge expense to maintain. In comparison, a ground-based coded VHF-receiver costs a few hundred dollars to build and can be constructed easily by anyone using online open-source instructions (e.g., sensorgnome.org). The positive responses by non-research participants and conservation-based NGOs in our survey show that hobbyists and amateur enthusiasts are a largely untapped resource that could assist in the initial construction of national or international animal biotelemetry tracking network, specifically filling in the spatial gaps between independent project-based receiver arrays.

## Conclusions

Long-term broad spatial scale studies across many individuals is required to better understand how our changing world is impacting upon animal populations and ecological communities. Greater collaboration between academia, government and non-government organisations will be required if we are to collect data over sufficiently broad spatiotemporal scales to address these issues with the limited funds available. This study informed us that amateur and professional ornithologists see value in nationally collaborative biotelemetry infrastructure for tracking birds, and if built, would use the network and share their data if appropriate agreements were in place.

## Supporting information

S1 Survey A survey to assess the opportunities and challenges of a national collaborative network for tracking the movement and survival of birds. (PDF)

S1 Fig Correlation matrix of individual mean responses to PCA components for survey questions relating to perceived benefits and challenges of a national network and data sharing. Lower matrix shows scatterplot of responses; diagonal shows distribution of responses, and upper matrix shows spearman rank correlation coefficients. * = p < 0.05, ** = p < 0.01, *** = p < 0.0001.
(PDF)

S1 Table Corrected Akaike Information Criterion scores (AICc), ΔAICc, and AICc weights for competing models (ΔAICc < = 2) looking at the effect of employment role (research or non-research), age group (early-, mid-, late-career), and years of biotelemetry experience (0, 1–9, 10+) on each component (response) variable relating to overall perceived benefits and concerns around the development of a national biotelemetry array. (PDF)

S2 Table Corrected Akaike Information Criterion scores (AICc), ΔAICc, and AICc weights for competing models (ΔAICc < = 2) looking at the effect of employment role (research or non-research), age group (early-, mid-, late-career), and years of biotelemetry experience (0, 1–9, 10+) on each component (response) variable relating to an individual’s potential interaction with a national biotelemetry network. (PDF)
