# Supplementary material for: Motivations and fears driving participation in collaborative research infrastructure for animal tracking
Source: PLoS One. 2020 Nov 20;15(11):e0241964. doi: 10.1371/journal.pone.0241964 (PMC7678966; doi:10.1371/journal.pone.0241964)
Supplement: S1 Survey — (PDF) [file pone.0241964.s001.pdf]

S1 Survey

A survey to asses the opportunities and challenges of a national collaborative network for tracking the movement and survival of birds.

We invite you to participate in a study examining the factors driving individual participation in a collaborative research network. The study is being conducted by Dr Tara Crewe and Dr Hamish Campbell at Charles Darwin University.

Participant Selection You have been selected to participate in this study because of your involvement and association with bird conservation, management, or research.

The Problem Banding recaptures are typically very low, making it difficult to use these data to understand population demographics. The development of technology that allows scientists to track animal movements throughout their annual life cycle is providing the context to better understand the factors driving population demographics, and how and when during the annual life cycle populations are most limited. However, GPS and satellite tracking devices remain too large to track most small-bodied animals, including many bird species.

The Solution In North America, the use of a collaborative autonomous radio-telemetry research network is improving recapture rates of marked animals and resulting in new and novel information on the movement behaviour and demographics of birds, bats and insects at fine to broad spatial and temporal scales. As an example, over 210,000 Blackpoll Warblers were banded in North America since 1960, but fewer than 60 (0.03%) were recaptured; in comparison, over 60% of radio-tagged Blackpoll Warblers have been recaptured by the biotelemetry network. This network, the Motus Wildlife Tracking System (www.motus.org), now covers much of eastern North America and parts of South America and Europe, and continues to expand globally. Such a network has the potential to fill knowledge gaps and contribute to improved conservation management plans for many of Australia's at-risk bird species.

Purpose of the Survey The aims of this study are:

1. To understand the Australian ornithological community's level of interest in a nationally coordinated biotelemetry network to track bird movements;

2. To identify perceived benefits and challenges of participating in and sharing data with such a network;

3. Develop a strategic plan that addresses identified challenges;

4. Present results in scientific journal articles and presentations.

Prize Money: At the end of the survey you will have the option to enter a draw for a $250 BCF gift certificate.

A survey to asses the opportunities and challenges of a national collaborative network for tracking the movement and survival of birds.

Terms and Conditions

Human Ethics This study has been approved by the Human Research Ethics Committee at Charles Darwin University. If you have concerns about the conduct of this study, complaints may be directed to the Ethics Administration Officer, Charles Darwin University, Darwin, NT 0909 AUSTRALIA (phone 8946 6923, email: cdu-ethics@cdu.edu.au).

Confidentiality and Disclosure of Information All questions will be answered anonymously and confidentially. E-mail addresses, if provided for the prize draw, will not be associated with responses. All electronic data will be secured on a password protected university computer and locked back-up drive.

Your consent Your involvement is this study is voluntary, and there is no obligation to participate. By submitting a filled survey, you are granting consent to use your answers in this research.

Inquiries Any inquiries about this survey or study can be directed to:

Dr. Tara Crewe at (tara.crewe@cdu.edu.au) or,
Dr. Hamish Campbell at (hamish.campbell@cdu.edu.au)

Possible benefits from participation in this study This survey will help determine the Australian ornithological community's level of interest in a national biotelemetry array to track bird movements. Concerns identified through this process can be addressed in the development of a strategic plan for implementation. Results from this study are likely to benefit those that are interested in participating in a network. However, we cannot and do not guarantee or promise that a network will be developed or that you will receive any benefits from this study.

Results from this study are also expected to be relevant to Australian telemetry in general because results can be compared with the benefits and concerns identified from other biotelemetry networks (e.g., fish telemetry).

Possible risks from participation in this study There are no identified risks to participation in this study. All information provided is non-identifiable and confidential. If you are not comfortable with answering some questions, you have the option to leave the question blank.

Individual Characteristics

1. What is your age group?

- ◯ 18-29
- ◯ 30-39
- ◯ 40-49
- ◯ 50-59
- ◯ 60-69
- ◯ 70+

2. Which of the following best describes your employment role?

- ◯ Undergraduate Student
- ◯ Higher Degree by Research Student
- ◯ Post-Doc/Research Assistant
- ◯ Faculty Position
- ◯ Industry/Government Scientist
- ◯ Environmental Consultant
- ◯ Conservation/Resource Manager

Other (please specify)

3. Do you hold an authority with the Australian Bird and Bat Banding Scheme?

- No
- C-class
- R-class
- A-class

4. Approximately how many years experience do you have using any type of biotelemetry for avian research?

- 0
- 1-4
- 5-9
- 10-20
- >20

5. Approximately how many birds have you tagged with telemetry devices?

- 0
- 1-20
- 21-40
- 41-60
- >60

Research Environment

6. In what country do you conduct your research?

- Australia

Other (please specify)

7. In which state/territory do you conduct your research? (Check all that apply)

- [ ] NT
- [ ] NSW
- [ ] WA
- [ ] QLD
- [ ] TAS
- [ ] SA
- [ ] ACT
- [ ] VIC

Other (please specify)

8. What is the nearest urban center to your study area(s)?

9. What environment(s) do you conduct bird research in? (Check all that apply)

- [ ] Coastal
- [ ] Inland
- [ ] Marine
- [ ] Migratory
- [ ] Shrubland
- [ ] Grassland
- [ ] Woodland
- [ ] Inland Waters
- [ ] Mangrove
- [ ] Savanna
- [ ] Urban
- [ ] Agricultural

Other (please specify)

10. Please indicate the avian species group(s) that your research encompasses (Check all that apply).

- [ ] Shorebirds
- [ ] Raptors
- [ ] Passerines
- [ ] Waterbirds
- [ ] Seabirds
- [ ] Megapods

Other (please specify)

11. What species do you currently work with?

Research Interests

12. What aspects of avian biology are you primarily interested in? (Check all that apply)

- [ ] Migration Ecology
- [ ] Movement Ecology
- [ ] Population Ecology
- [ ] Behavioural Ecology
- [ ] Social Behaviour
- [ ] Evolutionary Ecology
- [ ] Landscape Genetics
- [ ] Habitat Associations
- [ ] Physiology
- [ ] Predator-prey Interactions

Other (please specify)

Perceived Benefits and Risks of a Nationally Coordinated Array

13. On a scale of 1 to 10, how would you rank the ability of a nationally coordinated research infrastructure to enhance the management and conservation of Australian birds?

No Enhancement	2	3	4	5	6	7	8	9	Great enhancement
○	○	○	○	○	○	○	○	○	○

14. Please score from 0 to 10 **each of the following** fields in terms of the ability of a nationally coordinated research infrastructure to inform and advance the field.

Won't inform the field	2	3	4	Will somewhat inform the field	6	7	8	9	Will greatly inform the field
○	○	○	○	○	○	○	○	○	○

Comments

15. Please score from 0 to 10 **each of the following** potential benefits of participating in a nationally coordinated research infrastructure.

	No benefit	2	3	4	Somewhat beneficial	6	7	8	9	Great benefit
Publication opportunities	○	○	○	○	○	○	○	○	○	○
Funding opportunities	○	○	○	○	○	○	○	○	○	○
Improved conservation and management of species	○	○	○	○	○	○	○	○	○	○
Collaboration opportunities	○	○	○	○	○	○	○	○	○	○
Increased knowledge of bird movements over broad spatial and temporal scales	○	○	○	○	○	○	○	○	○	○
Reduced operational costs	○	○	○	○	○	○	○	○	○	○
Other (please specify in comments)	○	○	○	○	○	○	○	○	○	○

Comments

16. Please score from 0 to 10 **each of the following** potential challenges of participating in a nationally coordinated research infrastructure.

	Not a challenge	2	3	4	Somewhat of a challenge	6	7	8	9	Great challenge
Concerns with sharing data	○	○	○	○	○	○	○	○	○	○
User fees	○	○	○	○	○	○	○	○	○	○
Cost of infrastructure maintenance	○	○	○	○	○	○	○	○	○	○
Ongoing technological advances in GPS will supersede the need for a telemetry network	○	○	○	○	○	○	○	○	○	○
Data reuse guidelines	○	○	○	○	○	○	○	○	○	○
Technological support (towers, tags)	○	○	○	○	○	○	○	○	○	○
Analytical support	○	○	○	○	○	○	○	○	○	○
Gaining government/granting agency support	○	○	○	○	○	○	○	○	○	○
Non-Australian organization as network host (e.g. Motus in Canada)	○	○	○	○	○	○	○	○	○	○
Other (please specify in comments)	○	○	○	○	○	○	○	○	○	○

Comments

17. Please score from 0 to 10 **each of the following** potential benefits of a nationally coordinated database for monitoring bird movement.

	Not a benefit	2	3	4	Somewhat of a benefit	6	7	8	9	Great benefit
Broader spatial/temporal scales of research	◯	◯	◯	◯	◯	◯	◯	◯	◯	◯
Data discoverability and reuse	◯	◯	◯	◯	◯	◯	◯	◯	◯	◯
Standardized data/metadata	◯	◯	◯	◯	◯	◯	◯	◯	◯	◯
Reduced operational costs	◯	◯	◯	◯	◯	◯	◯	◯	◯	◯
Safe data storage/increased data permanence	◯	◯	◯	◯	◯	◯	◯	◯	◯	◯
New research perspectives	◯	◯	◯	◯	◯	◯	◯	◯	◯	◯
Collaboration opportunities	◯	◯	◯	◯	◯	◯	◯	◯	◯	◯
Other (please specify in comments)	◯	◯	◯	◯	◯	◯	◯	◯	◯	◯

Comments

18. Please score from 0 to 10 **each of the following** potential concerns with sharing bird detection data within a nationally coordinated database.

	Not a concern	2	3	4	Somewhat of a concern	6	7	8	9	Great concern
Lack of incentive/reward for sharing (e.g. co-authorship, proper citation)	◯	◯	◯	◯	◯	◯	◯	◯	◯	◯
Data reused without permission	◯	◯	◯	◯	◯	◯	◯	◯	◯	◯
Inappropriate reuse/interpretation of data	◯	◯	◯	◯	◯	◯	◯	◯	◯	◯
Non-Australian organization as database host (e.g. Motus in Canada)	◯	◯	◯	◯	◯	◯	◯	◯	◯	◯
Sensitivities around species locations	◯	◯	◯	◯	◯	◯	◯	◯	◯	◯
Time to publish before reuse	◯	◯	◯	◯	◯	◯	◯	◯	◯	◯
Other (please specify in comments)	◯	◯	◯	◯	◯	◯	◯	◯	◯	◯

Comments

19. What proportion of data contributed to a manuscript should command co-authorship?

0 50 100

20. If there were strict guidelines around data sharing, would you be more inclined to be part of a nationally coordinated biotelemetry network?

◯ Yes

◯ No

◯ Maybe

21. Please score from 0 to 10 **each of the following** for the level of interaction you may have with a nationally coordinated biotelemetry network.

	Unlikely	2	3	4	Somewhat likely	6	7	8	9	Very likely
Deploy transmitters on a single species										
Deploy transmitters on multiple species										
Deploy receivers in one location										
Deploy receivers across > 1 locations										
Undertake meta-analysis of the database										
Develop new modelling and analysis										
Apply movement data to landscape information										
Provide open access to the data										
Contribute to receiver software development										
Apply movement data to physiological information										
Other (please specify in comments)										

Comments

Wrapping up

22. What species do you think would be good candidates to make a start at tracking using a nationally coordinated research network? Why this species?
