# Supplementary figures and images for: Motivations and fears driving participation in collaborative research infrastructure for animal tracking
Source: PLoS One. 2020 Nov 20;15(11):e0241964. doi: 10.1371/journal.pone.0241964 (PMC7678966; doi:10.1371/journal.pone.0241964)

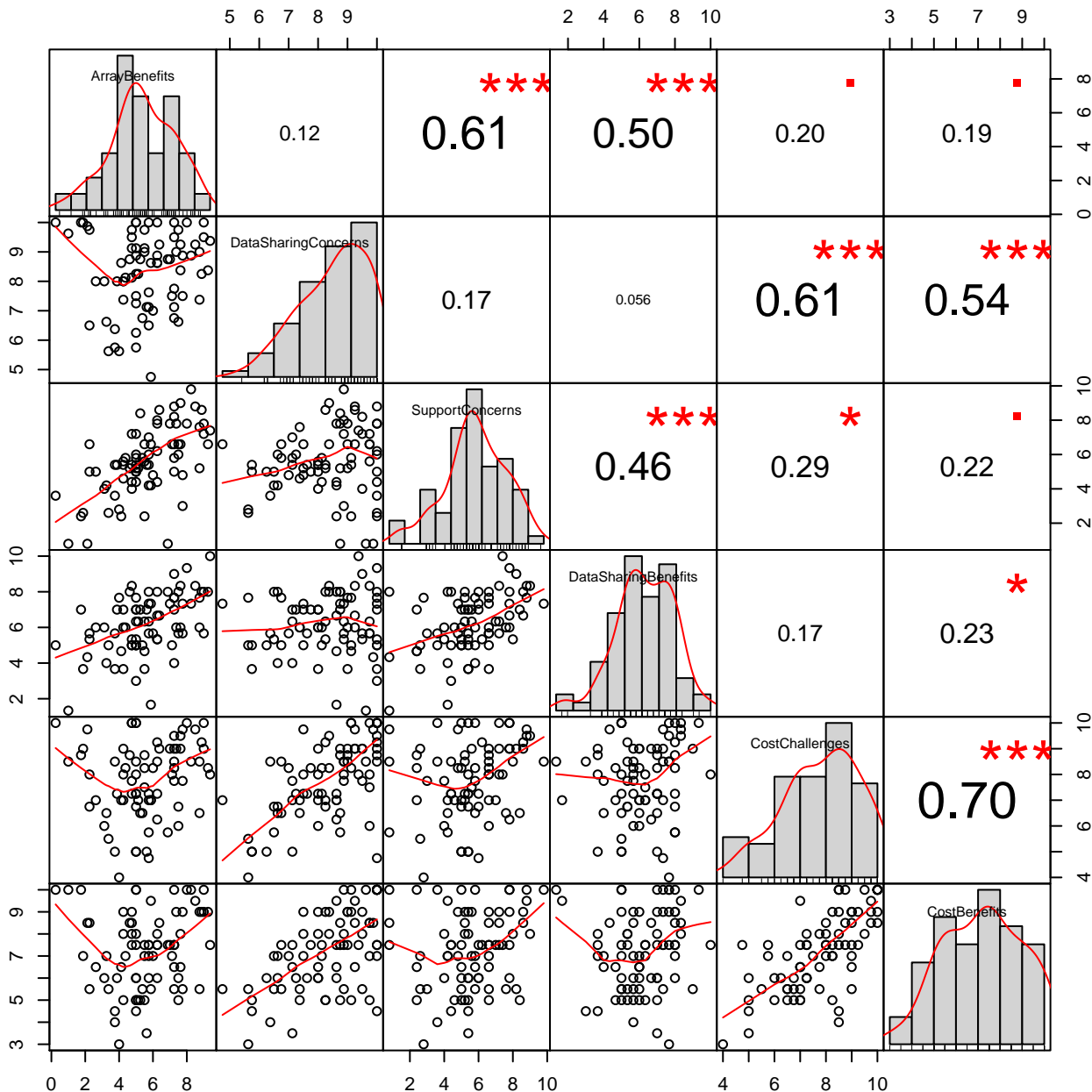

Supplement: S1 Fig — Lower matrix shows scatterplot of responses; diagonal shows distribution of responses, and upper matrix shows spearman rank correlation coefficients. * = p < 0.05, ** = p < 0.01, *** = p < 0.0001. (PDF) [file pone.0241964.s002.pdf]
