# Supplementary material for: Motivations and fears driving participation in collaborative research infrastructure for animal tracking
Source: PLoS One. 2020 Nov 20;15(11):e0241964. doi: 10.1371/journal.pone.0241964 (PMC7678966; doi:10.1371/journal.pone.0241964)
Supplement: S1 Table — (PDF) [file pone.0241964.s003.pdf]

S1 Table. Corrected Akaike Information Criterion scores (AICc), ΔAICc, and AICc weights for competing models (ΔAICc <= 2) looking at the effect of employment role (research or non-research), age group (early-, mid-, late-career), and years of biotelemetry experience (0, 1-9, 10+) on each component (response) variable relating to overall perceived benefits and concerns around the development of a national biotelemetry array.

Component	Predictors	k	AICc	ΔAICc	AICcWt	LL
Network Benefits	Employment role	3	337.75	0.00	0.33	-165.71
	Age group	4	337.82	0.07	0.32	-164.64
	Age group + Employment role	5	339.56	1.81	0.13	-164.37
	Biotelemetry experience	4	339.62	1.87	0.13	-165.54
Data Sharing Benefits	Employment role	3	305.46	0.00	0.41	-149.57
	Biotelemetry experience	4	306.70	1.24	0.22	-149.07
	Age group	4	307.07	1.61	0.19	-149.26
Data Sharing Concerns	Biotelemetry experience	4	264.34	0.00	0.53	-127.90
	Employment role + Biotelemetry experience	5	266.18	1.84	0.21	-127.67
Support Concerns	Employment role	3	331.18	0.00	0.59	-162.43
Financial Benefits	Biotelemetry experience	4	311.67	0.00	0.50	-151.56
	Employment role + Biotelemetry experience	5	313.49	1.82	0.20	-151.33
Cost Concerns	Employment role	3	287.30	0.00	0.49	-140.49

Biotelemetry experience	4	289.01	1.72	0.21	-140.23
