# Supplementary material for: Motivations and fears driving participation in collaborative research infrastructure for animal tracking
Source: PLoS One. 2020 Nov 20;15(11):e0241964. doi: 10.1371/journal.pone.0241964 (PMC7678966; doi:10.1371/journal.pone.0241964)
Supplement: S2 Table — (PDF) [file pone.0241964.s004.pdf]

S2 Table. Corrected Akaike Information Criterion scores (AICc), ΔAICc, and AICc weights for competing models (ΔAICc <= 2) looking at the effect of employment role (research or non-research), age group (early-, mid-, late-career), and years of biotelemetry experience (0, 1-9, 10+) on each component (response) variable relating to an individual's potential interaction with a national biotelemetry network.

Component	Parameter	k	AICc	ΔAICc	ModelLik	AICcWt
Network development	Age group	4	364.01	0.00	1.00	0.24
	Biotelem. Experience	4	364.07	0.06	0.97	0.23
	Age group + Biotelem. Experience	6	365.06	1.05	0.59	0.14
	Employment role	3	365.26	1.25	0.54	0.13
	Age group + Employment role	5	365.90	1.89	0.39	0.09
Data collection	Biotelem. Experience	4	362.28	0.00	1.00	0.57
